# Harveian Society of London

**Published:** 1888-03

**Authors:** 


HARVEIAN SOCIETY OF LONDON.

*The British Medical Journal* says of the Harveian Society of London : The annual general meeting and *conversazione* of this society took place on Thursday, January 19, at the Marlborough Rooms, Regent street. A business meeting for the election of officers for the ensuing year and for the usual complimentary votes of thanks to the retiring officials was held in the lower room ; and the retiring president, Mr. Edmund Owen, delivered an address which was heard with close attention by a large audience Dealing chiefly with the subject of medical education from the point of view of a hospital teacher, Mr. Owen pointed out with considerable force the disadvantages under which students of medicine must labor so long as the preliminary subjects continue to be a part of the hospital course, and showed how impossible it has become for any but the most brilliant to acquire real knowledge, owing to the constant demand made upon his time by periodical examinations which leave no interval in which to assimilate the knowledge thus forced into the mind. He criticised somewhat strongly certain recent enactments of the University of London relating to students failing to obtain honors, which he considered were more advantageous to the examiners than to the students themselves. At the close of the address, which was warmly applauded, he introduced the president for the ensuing year, Mr. W. Sedgwick, who briefly returned thanks for his election on taking the chair.

*Treatment of Loose Cartilage in Knee-Joint.*—Mr. HERBERT ALLINGHAM read a case of suture of the internal semilunar cartilage of the knee to the head of the tibia. The patient was a man, aged 35, who had been constantly laid up by slipping of the internal fibro-cartilage of the knee. An incision two inches long was made, with its centre over the cartilage. The knee-joint was opened, and a strong catgut passed through the fibro-cartilage and the periosteum of the upper end of the tibia. The joint was washed out with carbolic lotion, and the synovial membrane united with deep catgut sutures ; the wound was then closed without drainage. The patient, who was shown to the society, made a good recovery, and can now follow his employment.

